# Parental Perceptions of Physical Therapy Use Among Children With Cerebral Palsy: A Meta-Synthesis

**DOI:** 10.1097/jnr.0000000000000692

**Published:** 2025-07-08

**Authors:** Maggie Dumsile Dlamini, Ying-Ju Chang, Zama Mkhonta

**Affiliations:** 1 Department of Nursing, College of Medicine, National Cheng Kung University, Tainan, Taiwan; 2 Department of Nursing, Eswatini Christian Medical University, Lomkiri, Eswatini; 3 Institute of Allied Health Sciences and Department of Nursing, College of Medicine, National Cheng Kung University, Tainan, Taiwan; 4 Department of Maternity, Raleigh Fitkin Memorial Hospital, Manzini, Eswatini

**Keywords:** physical therapy, children with cerebral palsy, caregivers, perception, meta-synthesis

## Abstract

**Background:**

Cerebral palsy (CP), the most common cause of childhood motor disability, is often associated with comorbidities such as epilepsy and spasticity. CP is a heterogeneous group of disorders attributed to the nonprogressive injury of the developing brain during fetal life or infancy affecting posture and movement. Physical therapy, the most important current intervention for CP, targets the relief of muscle stiffness, reduction of perceived pain, and improvement of patient mobility.

**Purpose:**

This study was designed to synthesize the qualitative evidence on the perceptions of parents regarding the utilization of physical therapy in the treatment of children with cerebral palsy.

**Methods:**

Four electronic databases, including CINAHL, Embase, OVID Medline, and ERIC, were searched for relevant qualitative studies in March 2023. The included studies were critically appraised using the Critical Appraisal Skills Program tool for qualitative research by 2 independent reviewers, and a content thematic approach was used to synthesize the qualitative findings.

**Results:**

The 8 studies published between 1990 and 2022 included in this review covered data from 150 participants. The 47 findings extracted from these studies were grouped into 11 subthemes and finally into the following 4 synthesized themes: (1) physical therapy is an essential treatment with many positive outcomes, (2) the success of physical therapy depends on realistic partnerships, (3) honest and organized communication flow is critical, and (4) key challenges in physical therapy include service delivery, personal and family adjustments.

**Conclusions:**

Overall, the parents in the included studies perceived physical therapy as an ideal treatment associated with many positive outcomes for their children with disabilities and as a source of hope for their children’s future. Notably, the parents required clear information on the goals of therapy from the outset and to be involved in all care planning to promote therapy compliance.

## Introduction

Cerebral palsy (CP) significantly impacts the individual sufferer, their family, and the community. CP is a nonprogressive neurodevelopmental disorder that causes abnormalities in muscle tone, movement, and motor skills due to the injury of the developing brain (Gulati & Sondhi, 2018; Jackson et al., 2011; Levitt & Addison, 2018). Secondary impairments associated with CP include spasticity, contractures, coordination disorders, seizures, and intellectual, speech, vision, and hearing impairments (Gbonjubola et al., 2023; Jackson et al., 2011). CP-related motor disabilities, ranging from mild to severe, affect a child’s participation in normal activities and growth and often require that parents assist in daily self-care routines (Patel et al., 2020; Wang et al., 2017).

Over the past 2 decades, the global prevalence of CP has stood around 3 per 1000 live births (Levitt & Addison, 2018; Wati et al., 2020), with a lower incidence in developed than developing countries (Gbonjubola et al., 2021). The incidence of CP is 2.0~2.5 cases per 1,000 live births in the United States and about 3.5–4.0 cases per 1,000 live births in developing countries (Gbonjubola et al., 2021). Moreover, the prevalence of CP is much higher in males than females (Marian et al., 2020).

Caregivers primarily manage CP at home and continue with management efforts into the patient’s adulthood and beyond (Vitrikas et al., 2020). Parents, the primary long-term caregivers of children with CP, take on additional responsibilities due to disability-specific demands, financial constraints, treatment regimens, and family relationships (Dlamini et al., 2023; Smith et al., 2015). CP has no cure, and primary therapeutic interventions include continuous physical, recreational, speech, occupational therapies, and medical treatments (Green & Gaebler-Spira, 2019). Managing multiple disabilities in children necessitates a multidisciplinary approach to improve their daily life situation and walking abilities (Levitt & Addison, 2018). Physical therapy (PT) is a rehabilitative health approach focused on improving mobility, reducing pain, and alleviating muscle stiffness in individuals with CP (Gbonjubola et al., 2021; McCoy et al., 2020). PT is crucial in the management of children with CP, with a focus on symptomatic, preventive, and supportive treatments to enhance movement, function, and potential development (Wati et al., 2020). The various treatment methods associated with PT include exercise, joint mobilization, massage, heat and ice applications, and electrical stimulation (McCoy et al., 2020).

Caring for children with disabilities can be stressful for parents. The goal of this review is to identify and synthesize the qualitative studies in the literature on parental perceptions regarding physical therapy use among children with CP to synthesize available evidence and make it more accessible to healthcare providers and researchers.

## Methods

### Study Design

This meta-synthesis study was registered in the International Prospective Register of Systematic Reviews (PROSPERO; protocol number: CRD42023465384), and review procedures were guided by the step-by-step guide on how to do systematic literature reviews in nursing by Bettany-Saltikov (2016). In addition, the review was conducted in compliance with Preferred Reporting Items for Systematic Review and Meta-Analysis (PRISMA) checklist guidelines (Page et al., 2021). Two independent reviewers evaluated the included studies using the Critical Appraisals Skills Program (CASP; Long et al., 2020). In addition, content thematic analysis was used to code, group, classify, and synthesize qualitative research findings to enhance their value, consistency, and explicit knowledge (Nowell et al., 2017; Thomas & Harden, 2008). Ethical approval was not required due to the meta-synthesis nature of this study.

### Eligibility Criteria

The review included studies published in the English language that: (1) employed a qualitative or mixed methods study design and (2) included parents of children with CP under 18 years old using PT. The “PICo” framework was employed to explain the eligibility criteria, with (P) indicating participants were parents of children with CP under 18 years old; (I) indicating the phenomena of interest to be the perceptions of these parents; and (Co) indicating the context to be the use of physical therapy by the children. No restrictions on year of study publication were set.

### Search Strategy

The review question was thoroughly searched through a systematic review of journals and databases such as PROSPERO and Google Scholar to ensure it had not been the focus of a prior investigation. Four electronic bibliographic databases were systematically searched for relevant qualitative articles published in English with the assistance of a professional librarian in March 2023. These databases included the Cumulative Index to Nursing and Allied Health Literature (CINAHL), Embase, OVID MEDLINE, and ERIC. Based on the review question, the MESH and Emtree term list were used to select the search terms, which included “parent,” “caregiver,” “family,” “perception,” “thinking,” “comprehension,” “physical therapy,” “rehabilitation,” “child,” “cerebral palsy,” “muscle spasticity,” “focused groups,” “semi-structured interview,” “qualitative research,” “phenomenology,” “grounded theory,” “ethnography studies,” and “mixed methods.” These were used in the search in various combinations with Boolean operators “OR” and “AND.”

### Search Results

**Figure 1:**
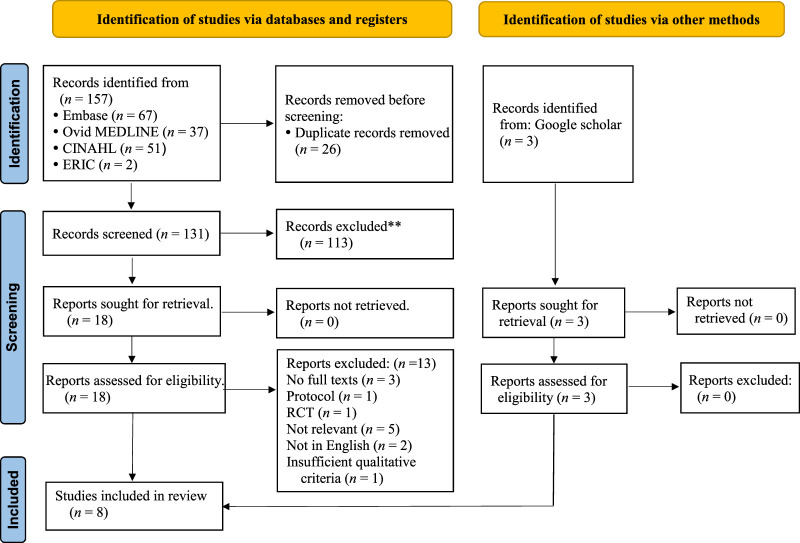
Flow Diagram of hte Screening Process

The initial literature search yielded 157 research papers from the 4 electronic databases, including Embase (*n* = 67), CINAHL (*n* = 51), OVID MEDLINE (*n* = 37), and ERIC (*n* = 2), as well as Google Scholar (*n* = 3). The eligibility and quality of these articles were assessed by 2 independent reviewers to optimize the selection process. Duplicate (*n* = 26), and irrelevant (*n* = 113) articles were removed. The full texts of the selected articles (*n* = 18) were downloaded from the databases or, if unavailable, requested from the corresponding authors through the library. After reviewing the full texts, those articles found to not meet the criteria (*n* = 13) were removed, leaving a final list of 8 articles that were included in the review. The reference lists in each of these 8 articles were searched to identify other articles potentially meeting the criteria that were missed during the database search. However, but none were identified. A PRISMA flow diagram of the screening process is displayed in Figure 1.

### Quality Appraisal of the Included Studies

The CASP checklist tool was used to appraise the quality of the included studies (Long et al., 2020). Two independent reviewers assessed the methodological validity of the appraisal, with mutual agreement reached without need of mediation by a third reviewer. The CASP checklist tool used 10 checklist questions for the included studies, with each answered with a Yes (Y) unclear (U), or No (N). Overall, the methodological quality of the 8 included studies was found to be high, with over two-thirds of the questions answered Yes (Y) in each study (average percentage of Yes answers: 82.5%; Table 1), indicating a low risk of bias (Carroll et al., 2012).

**Table 1:** Methodological Quality of the Included Studies Using the Critical Appraisals Skills Program Tool Study Checklist (N = 8)

Article	Q1	Q2	Q3	Q4	Q5	Q6	Q7	Q8	Q9	Q10	% of Yes
Answers											
1. Hinojosa, 1990	Y	Y	Y	Y	Y	U	U	Y	Y	Y	80
2. Christy et al., 2010	Y	Y	Y	U	Y	U	U	Y	Y	Y	70
3. Wiart et al., 2010	Y	Y	Y	Y	Y	U	U	Y	Y	Y	80
4. Egilson, 2011	Y	Y	Y	Y	Y	U	U	Y	Y	Y	80
5. Morgan & Tan, 2011	Y	Y	Y	U	Y	Y	Y	Y	Y	Y	90
6. Pereira Domenech et al., 2016	Y	Y	Y	Y	U	U	Y	Y	Y	Y	80
7. Perez-de la Cruz & Ramirez, 2020	Y	Y	Y	Y	Y	U	Y	Y	Y	Y	90
8. Chaovalit et al., 2022	Y	Y	Y	Y	Y	U	Y	Y	Y	Y	90

*Note.* Q1. Was there a clear statement of the aims of the research? Q2. Is a qualitative methodology appropriate? Q3. Was the research design appropriate to address the aims of the research? Q4. Was the recruitment strategy appropriate to the aims of the research? Q5. Was the data collected in a way that addressed the research issue? Q6. Has the relationship between the researcher and participants been adequately considered? Q7. Have ethical issues been taken into consideration? Q8. Was the data analysis sufficiently rigorous? Q9. Is there a clear statement of findings? Q10. How valuable is the research? Appraisal results: Yes (Y): Unclear (U), No (N); Summary of results: More than 2/3 of the questions of each study were answered yes and the average percentage of overall studies was 82.5%, indicating a low risk of bias.

### Data Extraction

A standardized collection form was used to extract relevant study design and results information from the primary research studies. This form collected information on author, year, studied country, number of participants, mean level of education, mean age, mean CP severity, study design, data collection, analysis, ethnicity, and findings. During data extraction, both reviewers worked together, reading each study to extract the key concepts. A summary of the results is presented in Tables 2, 3.

**Table 2:** Data Extracted From the Included Studies (N = 8)

Article Code	Author, Year, and Country	No. of Participants, Age and Level of Education	Children’s Age and CP Severity	Study Design	Data Collection and Analysis	Ethnicity	Findings
A1	Hinojosa, 1990, USA	*N*=8 (mothers) Participants ages not recorded Educational level not recorded	Children <5 years Severity Quadriplegic (*n*=4)Diplegic (*n*=2)Hemiplegic (*n*=2)	Exploratory ethnographic designs	Nonstructured interviews/constant comparative analysis	Diverse ethnicity • Caucasian (*n*=2) • Black (*n*=3) • Black Jamaican (*n*=1) • Caucasian English (*n*=1) • Caucasian English French (*n*=1)	1. What home programs? 2. If I only had a 25 hr day 3. We together 4. What does daddy do? 5. The long shadow 6. Therapy, therapy, therapy 7. The third parent 8. The roller coaster
A2	Christy et al., 2010, England	*N*=5 Mothers (*n*=4) Fathers (*n*=1) Participants ages not recorded Educational level High school diploma (*n*=1) College degree (*n*=3) Graduate degree (*n*=1)	Children <12 years Severity GMFCS level III (*n*=3) GMFCS level 1 (*n*=2)	Phenomenological designs	Semistructured interviews/constant comparative approach	White (*n*=4) African American (*n*=1)	1. The intense program improved motor function 2. The intense program improved confidence and independence 3. The intense program was a stressor to the family life during the program but allowed a time of rest and no therapy between sessions 4. The intense program encouraged and enabled participation in community 5. The intense nature of the program caused fatigue but enabled the perceived rapid attainment of functional goals
A3	Wiart et al., 2010, Canada	*N*=39 Mothers (*n*=34) Fathers (*n*=5) Participants ages not recorded Educational level not recorded	Children aged 2–17 years The severity of CP was not recorded	Descriptive study designs	Semistructured interviews/thematic analysis	Not reported	1. Movement as the means to functional success 2. Physical health and fitness are important therapy goals 3. The importance of leading happy, fulfilling lives and being accepted by others 4. We can’t do it all; balancing therapy with the demands of everyday life 5. Shifting roles and responsibilities in goal setting
A4	Egilson, 2011, Iceland	*N*=17 Mothers (*n*=14) Fathers (*n*=3) Participants’ ages ranged from 40 to 60 years Educational level not recorded	Children aged 7–13 years The severity of CP was not recorded	Qualitative study designs	Open interviews/thematic analysis	Icelandic	The role of the therapist 1. Monitoring the use of assistive devices 2. Providing practical information and advice 3. Improving physical function or promoting participation Service location and arrangements 1. Therapy within the local community 2. Service coordination at a national level Characteristics of good service 1. The family at the forefront 2. Help towards self-sufficiency 3. Professional behavior 4. Coordinating and preparing for the future
A5	Morgan & Tan, 2011, Cambodia	*N*=24 Mother (*n*=20) Fathers (*n*=4) Participants ages ranged from 18 to 55 years Educational level not recorded	Children aged 3–12 years Severity Hemiplegia (*n*=2) Diplegia (*n*=3) Quadriplegia (*n*=17)	Qualitative study design	Semistructured interviews/thematic analysis with NVivo software	Khmer	1. Collaborative partnerships 2. Information exchange 3. Respectful care
A6	Pereira Domenech et al., 2016, Brazil	*N*=11 Mothers (*n*=11) The participant’s mean age was 35.64 (± 15.10) years Educational level not recorded	Children aged 5 years and above Severity Quadriplegia (*n*=4) Diplegia (*n*=4) Hemiplegia (*n*=3)	Descriptive/exploratory study	Semistructured interview/Bardin´s content analysis	Latinos	1. Indispensable treatment for children diagnosed with cerebral palsy 2. Physical therapy helps to prevent complications, sequelae, and disabilities 3. Physical therapy helps children with cerebral palsy acquire independence and autonomy 4. Importance of continuing with therapy at home 5. Mothers compliance with the recommendations of physical therapists 6. Help from other family members 7. Stopped therapy at home because they were not advised to do so
A7	Perez-de la Cruz & Ramirez, 2020, Bolivia	*N*=27 Mothers (*n*=27) Participants’ ages ranged from 18 to 50 years Educational level No (*n*=8) Basic (*n*=7) Secondary (*n*=9) Professional (*n*=3)	Children aged 0–5 years The severity of CP was not recorded	Interpretive phenomenological designs	Semistructured interviews/thematic analysis	Hispanic	1. Participation 2. Trust and understanding 3. Benefits of therapy for the child 4. Improvements and benefits for family 5. Attention by staff 6. Negative aspects of therapy 7. Training
A8	Chaovalit et al., 2022, Thailand	*N*=19 Mothers (*n*=17) Fathers (*n*=2) Participants’ mean age mean age 39 years 6 months (*SD* = 8 years 4 months) Educational level High school (*n*=4) Diploma (*n*=5) Degree (*n*=10)	Children <14 years Severity of CP GMFCS level V (*n*=11) GMFCS level III (*n*=8)	Interpretive descriptive designs	Semistructured interviews/thematic analysis	Not reported	1. All caregivers saw positive changes in their children 2. Seeing positive changes gave caregivers hope that their child could develop with further training 3. The sit-to-stand program was feasible to complete at home

*Note.* CP = cerebral palsy; GMFCS = Gross Motor Function Classification System.

**Table 3:** Grouping and Coding Table (Findings, Subthemes, and Synthesized Themes)

Finding (Article Code)	Subtheme	Synthesized Theme
1. Therapy, therapy, therapy (A1) 2. The intense program improved motor function (A2) 3. Movement as the means to functional success (A3) 4. Physical health and fitness are important therapy goals (A3) 5. Physical therapy helps children with cerebral palsy acquire independence and autonomy (A6) 6. Benefits of therapy for the child (A7)	1. Physical therapy improves functional movement and development	1. Physical therapy is an essential treatment with many positive outcomes
1. The intense program encouraged and enabled participation in community (A2) 2. Monitoring the use of assistive devices (A4) 3. Improving physical function or promoting participation? (A4)	2. Physical therapy enables participation in age-related activities	—
1. Therapy is an indispensable treatment for children diagnosed with cerebral palsy (A6) 2. Physical therapy helps to prevent complications, sequelae, and disabilities (A6) 3. Mothers recognize the importance of continuing with therapy (A6) 4. Follow the recommendations of physical therapists (A6)	3. Physical therapy is essential and requires compliance	—
1. The intense program improved confidence and independence (A2) 2. Trust and understanding (A7) 3. All caregivers saw positive changes in their children (A8) 4. Seeing positive changes gave caregivers hope that their child could develop with further training (A8)	4. Ongoing physical therapy helps caregivers develop confidence and ensure great hope for the child’s future	—
1. What home programs? (A1) 2. The family in the forefront (A4) 3. Help towards self-sufficiency (A4) 4. Professional behavior (A4) 5. Collaborative partnerships (A5) 6. Respectful care (A5) 7. Participation (A7)	1. Mutual collaboration and supportive care	2. The success of physical therapy depends on realistic partnerships
1. We together (A1) 2. What does Daddy do? (A1) 3. The third parent (A1) 4. Help from other family members (A6) 5. Improvements and Benefits for the Family (A7) 6. Attention by staff (A7)	2. Joint relationships with the support system	—
1. The roller coaster (A1) 2. Shifting roles and responsibilities in goal setting (A3) 3. Providing practical information and advice (A4) 4. Information exchange (A5) 5. Mothers didn’t carry out exercises at home because they were not advised (A6) 6. Training (A7)	1. Information sharing about therapy	3. Honest and organized communication flow is critical
1. The importance of leading happy, fulfilling lives and being accepted by others (A3) 2. We can’t do it all; balancing therapy with the demands of everyday life (A3)	2. Self-acceptance of disability and setting priorities	—
1. Therapy within the local community (A4) 2. Service coordination at a national level (A4) 3. Coordinating and preparing for the future (A4) 4. Negative aspects of therapy reported by mothers (A7)	1. Administrative issues, and unavailable and poorly coordinated therapies	4. Key Challenges in physical therapy include service delivery, personal and family adjustments
1. If I only had a 25 hr day (A1) 2. The intense nature of the program caused fatigue but enabled the perceived rapid attainment of functional goals (A2) 3. The sit-to-stand exercise program was feasible to complete at home (A8)	2. Tiresome but adaptive	—
1. The long shadow (A1) 2. The intense program was a stressor to family life during the program but allowed a time of rest and no therapy between sessions (A2)	3. Alteration of family life, roles, and responsibility	—

*Note.* Themes and subthemes from the included studies were moved to a table to commence coding and grouping of the text. Codes were assigned to the articles using the letter A and a number.

### Data Synthesis

The qualitative research findings were assembled using a standardized collection form. The findings of the included studies were analyzed using content analysis, a qualitative method, to organize and interpret the collected data for realistic conclusions (Bengtsson, 2016). Themes and subthemes from the included studies were entered into a table to facilitate text coding and grouping. A code (the letter A followed by a number) was assigned to each article. The process involved assembling and grouping findings based on their similarities in meanings to create a set of statements representing that combination. Subthemes were generated from the grouped findings based on similar meaning (Thomas & Harden, 2008). The researchers synthesized the subthemes to generate themes based on parental perceptions of physical therapy use among children with CP, forming a comprehensive basis for evidence-based practice. In light of the importance of preserving meaning from original texts during data extraction (Noyes et al., 2019; Yadav, 2022), the researcher used direct quotes from primary studies and the interpretations of the original authors used in data synthesis to ensure the authenticity and originality of the findings.

## Results

### Characteristics of the Included Studies

Eight research studies published between 1990 and 2022 were included in the review. These primary studies were conducted in both developing and developed countries, including Thailand, England, Iceland, the United States, Cambodia, Brazil, Bolivia, and Canada. The participants (*n* = 150) in these primary studies and were all caregivers (mostly mothers) of children with CP under 18 years old undergoing PT. The children in these studies had been diagnosed with varied levels of CP severity. Three of the studies did not record the CP severity, and only five of the studies reported participant age ranges. Participants represented a diverse range of ethnicities, although two of the studies did not report participant ethnicity information. Although education is known to enhance cognitive abilities and foster critical thinking, which in turn influences perceptions, only three of the studies recorded the educational level of participants, which ranged from no formal education to a bachelor’s degree. The included studies used qualitative research (*n* = 2), phenomenological (*n* = 2), interpretive descriptive (*n* = 2), exploratory ethnographic, and descriptive exploratory (*n* = 1) designs, with all employing a series of face-to-face interviews for data collection that concluded with data saturation. In terms of analyzing collected data, thematic data analysis (*n* = 5), constant comparative analysis (*n* = 2), and Bardin’s content analysis (*n* = 1) were used in the included studies. During the review process, two of the studies (Hinojosa, 1990; Wiart et al., 2010) included both PT and occupational therapy (OT) but limited the synthesis to PT findings due to their primary aim being to evaluate and synthesize parental perceptions related to PT and the incompatibility with this aim of including OT findings. Furthermore, in the 2 studies that addressed PT and OT, data on the 2 therapies were generally reported separately. Due to the heterogeneity in how OT and PT were reported and analyzed, synthesizing data from both therapies may have diluted the focus and clarity of the review findings. While ensuring rigorous and coherent synthesis in line with PT being the single focus of this review, these 2 dual PT/OT studies were included in the data analysis. The researchers ensured all of the included studies complied with research ethical guidelines for research involving human subjects.

### Synthesized Findings

The 47 discrete findings extracted from the 8 included studies were first grouped into 11 subthemes and then into 4 synthesized themes as shown in Table 3. The synthesized themes include: (1) physical therapy is an essential treatment with many positive outcomes, (2) the success of physical therapy depends on realistic partnerships, (3) honest and organized communication flow is critical, and (4) key challenges in physical therapy include service delivery, personal and family adjustments.

#### Theme 1: Physical therapy is an essential treatment with many positive outcomes

PT is a crucial treatment for children with disabilities that enhances mobility, physical fitness, participation in age-related activities, and self-care task efficacy and promotes positive changes in a child’s development, including independent movements and fine motor activities. Therapy requires compliance from parents and children to achieve treatment success. In this review, the participants were found to glean hope for the future of their children from the positive aspects of PT.

Subtheme 1: Physical therapy improves functional movement and development

The participants generally reflected the belief that PT is an effective treatment program for the development of their children and that it improves both strength and function, minimizes/prevents the worsening of musculoskeletal problems, reduces the risk of contractures, and improves fine motor activities. However, although most of the participants valued therapy as a valuable treatment, some of the participants held different opinions and believed their children would never be normal.

*“It´s like, physical therapy has helped him a lot, both with the walking issue and with the motor issue, and also with socialization, he was a very shy child, … so nowadays he goes downstairs by himself because he knows it’s important for him.”* (Pereira Domenech et al., 2016).

*“So, I know if we do that for typical kids, then to me it is ludicrous to [think] every special-needs kid with CP has got to get down and crawl. He is not a perfect picture. The damage is done. These kids are not going to be typical. Why do we have to say that ‘normal’ is the only way they should be?”* (Wiart et al., 2010).

Subtheme 2: Physical therapy enables participation in age-appropriate activities

The participants valued the role of PT assistive devices in encouraging their children to engage in age-appropriate activities such as swimming and wheelchair basketball, which improved their motor function and self-esteem and stimulated hidden abilities. However, limits on the availability of assistive devices were identified as a key challenge.

*“If he wasn’t going to Lakeshore [Foundation], I would probably have him in physical therapy … but that is therapy. (Sam) plays wheelchair basketball, swims, jumps on the trampoline and stuff.”* (Christy et al., 2010).

*“Tumi’s just switched physical therapists and the one who took over is just great. He showed initiative in increasing his number of visits from 2 times a week to 3. He uses a new exercise program that has been so easy for us parents to follow.”* (Egilson, 2011).

Subtheme 3: Physical therapy is essential and requires compliance

The participants viewed PT as a crucial treatment for children with CP that requires compliance. PT contributes to motor development by improving functionality, stimulating nerves, and preventing disability-related complications. The participants reported complying with therapy protocols and the therapist’s recommendations and continued the program at home.

*“I believe physical therapy is of crucial importance for him; he cannot be without it.”* (Pereira Domenech et al., 2016).

*“I do follow some recommendations they gave me, I try and do it with her, I do whatever I can, and we have already noticed a great improvement in her.”* (Pereira Domenech et al., 2016).

Subtheme 4: Ongoing physical therapy helps caregivers develop confidence and ensure great hope for the child’s future

Commitment to continuing PT has many developmental gains for children with CP. Due to PT, the children in these studies were able to try new functional skills such as taking steps, eating, walking, and accomplishing other self-care tasks. Their children’s progress gave the participants courage and satisfaction and promoted adherence to therapy. The participants perceived PT as effective and rewarding with many objective and positive outcomes that encourage children to train to accomplish even more complex tasks.

*“I see that he is improving and, over time, the changes are noticeable; they make him work longer. He does a lot of exercises and is active.”* (Perez-de la Cruz & Ramirez, 2020).

*“I think this PT exercise has made me more interested in changing my child’s development. Just 6 weeks of training gave her more confidence in doing various activities at home, such as eating, and other self-care tasks.”* (Chaovalit et al., 2022).

#### Theme 2: The success of physical therapy depends on realistic partnerships

PT is central to the effective management of children with CP-related disabilities and requires therapists to purposefully partner with parents and act as family confidants to enable shared care responsibilities. The role of therapists is to monitor, advise, and promote participation, improving physical function and preventing complications. Mutual respect, support, and involvement in the therapy goal-setting process have been shown to have positive therapeutic outcomes in children with disabilities.

Subtheme 1: Mutual collaboration and supportive care:

The participants had a generally low level of knowledge regarding therapy goals and thus may easily feel frustrated. Notably, they felt being fully involved in therapy implementation and arrangements was an important aspect of raising their children with CP. Thus, parents should collaborate with therapists, participate in informed decision-making, and plan for their children’s care services, ultimately being able to choose on their own the best courses of action. Therapists were preferred by the participants for their expertise, guidance, and supportive care, which can provide them with informed decisions and effective treatments.

*“We are the ones at home with our child. We know what she can do and what her leg is like. The staff only see and get to know these children outside of the home, whereas we live with them daily. I think the staff will understand only after they ask the mother what the child is like at home with their family and what their needs and problems are. If we only ever see the child in the Centre, we don’t know everything.”* (Morgan & Tan, 2011).

*“The reason that I take my child to the Centre is that the staff pay attention to the patients, and never do anything that upsets us. I have noticed that they use friendly and funny words with children. The friendliness of the Centre’s staff and the attention they show to my child are the reasons I take my child to receive the services there.”* (Morgan & Tan, 2011).

Subtheme 2: Joint relationships with the support system

The therapist’s passion, warmth, and positive attitude can improve children with CP, while family support, particularly from supportive fathers, can enhance therapy adherence through playing and interacting with and accompanying their children during therapy sessions. Family support for children with CP varied among the participants, with some shouldering the burdens of home treatment alone. Long-lasting relationships with therapists and other parents of children with CP represent essential sources of support. The degree to which the needs of each child are met depends largely on each parent-child relationship.

*“One thing I realized - The friends I had before having Carrie will always be my friends. But, after having Carrie, I had to get new friends who understood the experience of being a mother of a handicapped child. This is why my new friends are parents of handicapped kids. Because this is going to be my life forever now, after having Carrie*.”(Hinojosa, 1990).

*“I do the majority of the hospitals and stuff with the twins because he has to work. But when I can’t, he’s there. And he helps in a million other ways.*”(Hinojosa, 1990)

*“As for the therapists, we have no complaints; they treat us well. Sometimes they call me if we are missing sessions because we don’t have money to come by public transport.”* (Perez-de la Cruz & Ramirez, 2020)

#### Theme 3: Honest and organized communication flow is critical

Effective communication about CP, its pathology, and therapy goals helped the participants and their children navigate future challenges and accept the disability. Collaborative therapy goal-setting between the therapists and participants enhanced childcare quality, providing a clear understanding of treatment options, goals, and knowledge of a child’s conditions as well as enabling acceptance and realistic future choices. Useful information sharing and coaching helped the participants continue with therapy exercises even at home.

Subtheme 1: Information sharing about therapy

By addressing therapy goals and uncertainties surrounding their children’s future, clear communication between the participants and therapists was identified as key to therapy adherence. Knowledge about available therapy options and therapist recommendations were instrumental in easing daily life concerns and opening up possibilities for the future care of their children. On the contrary, minimal/inadequate information sharing may lead to noncompliance with recommended treatments.

*“When you don’t get a lot of goals back from how the therapist communicates with me, you have no idea where you are going with her. I don’t know! I have no idea what I am doing. You have to know that. Please don’t think Mom is an expert. I live with her, but I don’t know what the possibilities are.”* (Wiart et al., 2010)

*“It’s important I find staff who can give me information that I can use, like the staff who strengthen my heart so that I can support my child. But the staff guide us well. They instruct us and strengthen our morale so we can support our child and don’t lose hope in our disabled child.”* (Morgan & Tan, 2011)

Subtheme 2: Self-acceptance of disability and setting priorities

Acceptance of their children’s disability was a fundamental factor beneficial to both family and child. Acceptance reduces worry, breaks societal barriers, makes people happy in spite of their disability, and allows them to live in the “normal” world. The children’s happiness regardless of their disability and enjoying family life was identified by the participants as more important than focusing on therapeutic activities.

*“These kids are not going to be typical. Why do we have to say that ‘normal’ is the only way they should be? To me it is crazy. My husband and I are practical. We wanted our daughter in a wheelchair at 3, because we wanted to announce to the world that she is different. She doesn’t communicate the way that you think she is going to. It was a social moment for us to go, please give us something that indicates to the world that ‘-Yes, she is special.’”* (Wiart et al., 2010)

#### Theme 4: Key Challenges in physical therapy include service delivery, personal and family adjustments

PT, despite its positive outcomes, was identified as having negative aspects such as being tedious, unavailable, and potentially changing family roles, posing a significant burden for families. The rehabilitative centers were situated far from where the participants and their children resided, posing significant travel challenges. Although therapy may sometimes be unavailable, uncoordinated, or limited due to restricted working hours. Accessible and adequate local community therapy is crucial for continuity of care. Thus, coordinating rehabilitative services at the national level is necessary. Although the studies included in this review were published across a time period of over 30 years, the participants reported experiencing similar challenges in physical therapy service delivery, including limited access to information, poor societal attitudes, inadequate healthcare policies, and poorly coordinated therapeutic approaches.

Subtheme 1: Administrative issues and unavailable and poorly coordinated therapies

PT is an essential treatment for children with CP. Thus, the participants expected it to be conveniently accessible to maximize their child’s participation. The participants advocated for therapy to be integrated into local health centers and rural schools, ensuring a holistic approach and proper staffing to guide parents through their care journey. Experiencing poorly coordinated therapy and therapist shortages contributed to low satisfaction with PT.

*“The therapy should just clearly be a part of everyday life as much as possible. It should be in the home, preschools, and schools where the child is.”* (Egilson, 2011).

*“There needs to be one person with a whole view over all the human developmental elements to connect the home, school, and other places where the child seeks services, help people prioritize, and push things along. This is necessary to guarantee a holistic approach, to ensure people are headed for the same goal.”* (Egilson, 2011).

Subtheme 2: Tiresome but adaptive

PT was perceived as a demanding and overwhelming task requiring coping strategies and imposing high stress levels on the participants and their families due to its exhaustive nature. Home-based therapy implemented with therapist training was deemed feasible.

*“I mean, I’m the one that has to do all the running round… I’m the one always running to therapy, always going to the doctor.”* (Hinojosa, 1990).

*“Traditional therapy I guess would just be a 30-minute blip and it’s hard to get as much in, and [the program] is a lot more intense.”* (Christy et al., 2010).

Subtheme 3: Alteration of family life, roles, and responsibility

Although having a child with CP participate in a PT program disrupts the family lifestyle, this therapy is beneficial overall. Increasing integration of their child into the family unit was made possible through the family’s development of coping skills. Also, older siblings were reported to assume parental roles and responsibilities for the child.

*“So many things were happening in my household because of Carrie. I didn’t realize that it was because of having this handicapped child… and how much pressure it put on everybody involved with her. And as time went on, I began to see that.”* (Hinojosa, 1990).

*“He (older brother) is like a father to him. He makes his breakfast in the morning before he goes to school, and he feeds him, sometimes. He fixes breakfast.”* (Hinojosa, 1990).

## Discussion

This review shed light on various aspects of parental perceptions of PT use in children with CP. The participants perceived PT as an essential treatment for children with disabilities, which resulted in many positive child developmental gains and PT being valued for its effectiveness. PT was identified as facilitating social, psychological, and physical recovery in children with CP by enabling their development of new functional skills and engagement in self-care activities using assistive devices. Therapists, experts in the therapeutic management of impairments, advise, monitor, and promote the participation of affected children in PT programs to improve their physical function and prevent future complications (Killingback et al., 2022).

The participants in this study perceived PT as crucial for their children’s motor and psychological development, social independence, and improved behavior and to the prevention of future complications. Gunel (2009) reported that PT minimizes the effects of children’s physical impairments and helps children with disabilities gain their independence. Positive PT leads to minimal therapy abandonment in children with CP, emphasizing the necessity of ongoing treatment despite caregivers’ high stress and uncertainty. Similar studies involving parents of children with CP showed that therapy offers numerous advantages (Almasri et al., 2018). PT promotes participation in community activities among children with disabilities and reduces caregiver strain, rewarding parents’ efforts. PT is crucial in managing CP, as it aids in strengthening muscles and enabling children to get on their feet again (Anttila et al., 2008).

PT is a crucial approach to treating children with physical disabilities, and the participants in this review recognized success as depending on compliance and adherence to therapist recommendations. The current literature identifies PT efficacy to be significantly affected by patient compliance with prescribed exercise protocols (Chan & Can, 2010). Some of the participants reported continuing home-based motor skills development treatment protocols, fearing noncompliance may harm development, while some only partially complied with protocols due to time constraints and/or fear of incorrect follow-up. This review revealed that parents of children with CP face a dilemma in physical therapy in terms of striking an appropriate balance between achieving long-term health and comfort and preventing unnecessary pain and stress. Resolving this dilemma will require a comprehensive approach that considers parents’ needs and perspectives and utilizes a diverse range of strategies to mitigate concerns. Parents should be empowered to voice concerns and preferences about therapy sessions and allowed to work with therapists to find effective and comfortable alternative approaches. Transparent, honest, and open communication lines and collaboration among therapists, parents, and children are crucial to addressing everyone’s concerns. Establishing a supportive and non-judgmental environment can foster better collaboration and problem-solving by allowing concerns to be freely expressed. Holistic support may involve connecting families with additional resources, support groups, or mental health services to ensure comprehensive care and well-being.

This review revealed the unique needs of children with CP and the need for personalized therapy plans that are flexible, adaptable, and responsive to individual participant needs and progress. While parents are quite often responsible for delivering multiple home therapy programs to children with disabilities (Alabdulqader et al., 2022), therapists can improve treatment compliance by creating personalized programs that involve both parents and children, respect available family resources, and provide positive feedback to build trust and maintain continuity (Seewoodharry et al., 2017). The ultimate goal is to foster a collaborative and supportive environment in which parents, therapists, and children work together to enhance the quality of life of the children, adjusting therapy plans based on individual needs and fostering open communication for optimal care. Collaboration helps set clear therapy goals, fostering teamwork and motivation through shared objectives and milestones. Therapists must conduct regular assessments to track participant progress and therapy effectiveness, involving feedback from both children and their parents to identify areas in need of improvement or adjustment.

Family-centered care may be effectively integrated into the care of children with CP, promoting mutual collaboration, and supportive care, and allowing adaptation to therapy services. The family-centered care approach is based on mutually beneficial partnerships between service providers, families, and children (Aydin & Nur, 2012). Therapists should support parental therapy adaptations and joint goal-setting to meet children’s developmental needs. The findings of this review support previous research indicating unsupported parents and children are more likely to exhibit low compliance and adherence to prescribed therapy exercises (Galil et al., 2001). Therapists provide a strong support system, foster long-lasting relationships, play a central role in the child’s life, and understand the child’s situation.

Although most of the participants perceived therapists as reliable sources of therapy-relevant information, some felt that therapists did not adequately involve them in planning and goal setting, as they were economical and did not involve them in planning and goal setting. In fact, few of the participants reported receiving relevant information about therapy. Parents seek a comprehensive understanding of treatment reasons and future care options, which may be enhanced through better information sharing and strengthening the relationship between service providers and patients (Clochesy et al., 2015). Limited information, poorly coordinated therapies, and the insufficient availability of therapies may interfere with therapy compliance and create low satisfaction among parents. Parents must be provided comprehensive education on cerebral palsy, physical therapy, and other available treatment options, empowering them to become active participants in their child’s therapy journey and to advocate for their needs effectively. The findings of this review suggest that well-coordinated therapy services and clear goals can assist families in their care journey and facilitate their children’s continued participation and adherence to therapist recommendations. These findings echo those of previous studies reporting that fragmented healthcare systems increase patient dissatisfaction with the healthcare provided (Stange, 2009).

Despite its positive outcomes, therapy is often a significant source of stress for families due to the fatigue and travel difficulties involved in making weekly therapy visits. Having a child with CP changes every family member’s daily life due to the involvement in that child’s holistic care (Ketelaar et al., 2008). Service providers should offer flexible care interventions that accommodate the needs of the family and others involved and promote the development of adaptive skills and coping strategies necessary for parents to assume additional responsibilities. Despite the various therapy-related stressors, PT is beneficial overall to children with disabilities and helps facilitate their rapid attainment of functional goals.

Encouraging and empowering children with CP to actively participate in the therapy can enhance therapy dialogue and ease the dilemma faced by parents through sharing experiences and perspectives and helping children advocate for themselves, helping parents and therapists better understand these children’s needs and challenges. This result not only promotes autonomy and self-advocacy but also offers valuable insights into the most effective approaches for children. Adopting a collaborative, individualized, and holistic approach to physical therapy use helps ensure the concerns and dilemmas faced by parents are effectively addressed, leading to better outcomes and improved quality of life for the child. In this review study, families with children with disabilities who encouraged their children to accept their disability were shown to lead fulfilling lives, make choices, and overcome societal barriers. Self-acceptance of disability and commitment to PT program participation reduce social anxiety and depression, and enable children with disabilities to live successful lives and engage positively with the world around them (Rostami et al., 2014). The participants in this review study were found to prioritize the happiness of their children over participation in therapeutic activities, wishing therapy could be provided in local health clinics and schools and that PT exercise videos would be available to facilitate PT being done in the home. The review focused on parental perceptions of PT use, and findings related to OT were not a primary focus. Future research could explore OT separately or its use in combination with PT for broader understanding and additional insight.

### Limitations

The review is affected by several limitations. The primary focused on PT excluded the consideration of occupational, recreational, and speech therapies, which are crucial in managing children with CP. Also, this review was limited to articles published in English, excluding the significant body of works on this subject published in other languages. Another limitation was the small number of regions covered in the included studies, which may limit its generalizability to other regions and cultures.

### Implications for Practice and Policy

Clinicians should make patient information on treatment goals and disease pathology more accessible to parents to improve therapy adherence and goal attainment. Also, therapists may provide parents with video recordings of exercise program demonstrations and explanations to promote the continuation of therapy exercises at home. Finally, policymakers should improve PT facility accessibility to the population, even in rural areas, to alleviate the travel burdens on families of children with CP.

### Recommendation for Future Research

A future review may be conducted to investigate parental perceptions of the other essential therapies, including occupational, recreational, and speech, used to manage children with CP to provide a comprehensive evidence base.

### Conclusions

The current qualitative evidence on parental perceptions of PT use among children with CP was synthesized in this review. In general, the parents viewed PT as an ideal treatment with numerous positive outcomes and expressed holding strong hope for their children’s future. Therapists must educate parents on therapy goals from inception and further involve them in the therapy planning process to promote compliance. Parents of children with disabilities should be encouraged and enabled to continue therapy exercises at home.
